# Update on Screening for Urological Malignancies

**DOI:** 10.5041/RMMJ.10318

**Published:** 2017-10-16

**Authors:** Azik Hoffman, Elizabeth E. Half

**Affiliations:** 1 Division of Urology, Department of Surgical Oncology, Princess Margaret Hospital, Toronto, ON, Canada; 2 Department of Gastroenterology, Rappaport Faculty of Medicine, Technion–Israel Institute of Technology, Haifa Israel; 3 Gastroenterology Institute, Rambam Health Care Campus, Haifa, Israel

**Keywords:** Cancer, screening, urological malignancy

## Abstract

Urological malignancies are a major source of morbidity and mortality in men over 40. Screening for those malignancies has a potential benefit of reducing both. However, even after more than two decades of screening for prostate cancer, the implications of most resulting information are still a matter of debate. Controversy extends over several aspects of prostate cancer screening programs, including age of onset, defining populations at risk, most appropriate intervals, as well as the optimal methods to be used for screening. The medical community is still divided regarding the effectiveness of prostate cancer-related death prevention and its benefits-to-harms ratio, reflecting an inconsistency regarding screening recommendations. Similarly, benefits of screening for urothelial and kidney tumors are yet lacking high-level evidence, although recent evidence supports screening of populations at risk. Clearly, the current era of evolving molecular and genetic biomarkers harbors the potential to change screening practice. In this paper, we review current guidelines as well as giving an update on new developments which might influence screening strategies in common urological malignancies.

## INTRODUCTION

Cumulatively, the most common urological malignancies (i.e. cancers of the prostate, bladder, and kidney) are the most common group of cancers in the Western world. Therefore, an effective screening program that reduces morbidity and mortality would be of importance. Recently, new molecular markers, as well as imaging studies, have been proposed to better detect cancer in urological organs. In addition, certain populations at risk have been better defined, and therefore a reconsideration of possible screening strategies in urology is warranted.^1^ This must address benefits and limitations, as well as the need to tailor screening protocols to populations at risk.

An effective screening program should consider several significant parameters:

1. The disease should be sufficiently prevalent in the population, or there should be a defined at-risk population.
2. The screening test must be sensitive and specific enough without additional morbidity.
3. The test must detect an early disease to be able to influence outcomes.
4. It should be cost-effective.^2^

## SCREENING FOR PROSTATE CANCER

Since the discovery of prostate-specific antigen (PSA), most reported data regarding screening in urology focus on prostate cancer. New data regarding prostate cancer screening may potentially be applicable to other urological cancers as well.

Prostate cancer is the most commonly diagnosed cancer in men and the seventh leading cause of male cancer deaths.^3^ The lifetime risk of being diagnosed with prostate cancer is 16%; however, only approximately 3% will die of it.^4^ Prostate cancer prevalence is rising with age, and can reach up to 60% in men at the of age of 80, as suggested by autopsy series, although most of these cancers are clinically insignificant.^5^ This is mainly due to the fact that most prostate cancers grow slowly, and men at these ages die of other causes. Prostate cancer survival is strongly related to the pathological Gleason grade and tumor local extension at the time of diagnosis. Although 5-year survival rates of patients with organ-confined or locally invasive disease is almost 100%, it reduces dramatically to approximately 30% if distant metastases are present at the time of diagnosis.^4^ An effective screening program which identifies localized tumors with aggressive features is therefore expected to reduce prostate cancer morbidities such as local and metastasis complications, treatment complications, and mortality. Originally, PSA was used to detect prostate cancer recurrence or progression, but later it became widely used for screening, as guidelines were issued accepting it as a screening tool.^6^,^7^ This led to increased detection of prostate cancer, mainly low-risk localized disease.^8^ Eventually, surgery and radiation therapy were increasingly utilized to cure these cancers, consequently leading to increased adverse events and morbidity.^9^–^12^

In the absence of sufficient data from randomized trials, prostate cancer screening has become a controversial issue.^13^ Two major randomized trials attempted to settle this controversy. The European Randomized Study of Screening for Prostate Cancer (ERSPC) reported minor survival benefit in favor of the screened population after 9 years.^14^ However, 48 new cases of prostate cancer would have to be diagnosed to prevent 1 cancer-related death. A subsequent report highlighted the potential harms of treatments, including erectile dysfunction, incontinence, and bowel dysfunction.^15^ Similarly, the Prostate, Lung, Colorectal, and Ovarian (PLCO) Cancer Screening Trial reported no benefit for screening using PSA and digital rectal examination after 10 years of follow-up.^16^ The levels of PSA may be elevated in men with prostate cancer due to the fact that PSA production in cancer cells is increased and PSA tends to leak more into the blood, preceding clinical disease by 10 years or longer.^17^–^19^ Serum PSA may also be elevated in benign prostatic conditions, and this should be considered when using PSA for screening. Additionally, overestimation of sensitivity and underestimation of specificity of the test is driven by the fact that men will not be biopsied at normal ranges of PSA unless having an abnormal digital rectal examination, making its performance assessment inaccurate.^20^ Also, since PSA often detects low-risk disease, performance status should be evaluated for clinically significant prostate cancer. Since the definition of significant prostate cancer findings on biopsy, i.e. pathological and clinical findings that might predict metastasis and subsequently lead to disease-related mortality, has shifted and recently been re-assessed, previous PSA-based screening programs might not accurately represent modern practice. Nevertheless, PSA screening often triggers biopsies of the prostate, most often performed by needle biopsy which has a false-negative rate of 10%–20%.^21^,^22^ Increasing the number of samples obtained increases the detection rate, yet some significant cancers might be missed.^23^,^24^ On the other hand, increasing the number of cores sampled leads to greater proportions of low-risk cancers detected. A review estimated that as much as 25% of prostate cancer lesions detected on prostate biopsy are smaller than 1.0 cm^3^ and cannot be the reason for the increase in PSA that triggered the biopsy.^25^ Therefore, any screening program should be evaluated based on ability to detect clinically significant cancers. Although no consensus is available regarding this, most reports refer to tumors with Gleason scores ≥7, adverse pathological patterns (such as cribriform), and increased tumor size (>0.5 cm^3^) to be at risk for progression.^26^ The traditional cut-off for an abnormal PSA level in the major screening studies has been 4.0 ng/mL.^27^–^30^ Screening studies best characterize PSA cut-off levels based on the proportion of men with an elevated PSA who eventually are diagnosed with cancer (positive predictive value, PPV). The PPV for a PSA over 4.0 ng/mL is estimated to be 30%.^27^,^31^,^32^ This increases to 42%–64% for PSA levels >10 ng/mL.^31^,^33^ On the other hand, one must consider that nearly 75% of cancers detected with PSA values between 4.0 and 10.0 ng/mL are organ-confined and potentially curable.^31^ The high false-positive rate for PSA levels lower than 10 ng/mL eventually leads to more biopsies. Although rare, the potential to miss prostate cancer in men with low PSA levels is also present. The Prostate Cancer Prevention Trial (PCPT) included men with PSA values within what is considered the normal range (up to 4.0 ng/mL). Among 675 men with PSA levels of 2–4.0 ng/mL, almost one-quarter were diagnosed with prostate cancer. Most of the cancers were clinically insignificant, but four patients did harbor potentially aggressive Gleason score ≥8 cancers.^34^ This suggests that there is no clear-cut definition for an abnormal PSA level. Any cut-off would involve a trade-off between sensitivity and specificity. Lowering the cut-off to 2.5 ng/mL, as suggested by some, would double the number of men defined as having abnormal levels while detecting cancers which may not become clinically significant, leading to overdiagnosis and overtreatment.^35^ Improving the ability of PSA to predict significant disease at values of ≤10.0 ng/mL is therefore warranted. Suggested PSA modifications, such as measuring velocity (change over time) and using age- and race-specific ranges, have been reported.^36^ A systematic review found no evidence to support the use of PSA velocity in screening.^37^,^38^ Another suggested approach is measuring a free-to-total ratio of PSA. A meta-analysis concluded that it is generally only clinically helpful at extreme values.^39^

Two recent methods have been evaluated to improve decision-making regarding additional sets of prostate biopsies when previous biopsy was negative but a significant prostate cancer is suspected. The first, *Prostate Health Index* (PHI), incorporates PSA derivates (total PSA, free PSA, and (-2)ProPSA). A meta-analysis estimated a specificity of 0.32 for the PHI.^40^ Area-under-the-curve (AUC) ranged from 0.70 to 0.77 for PHI, suggesting improved performance compared to PSA. Based on the publications, the American National Comprehensive Cancer Network (NCCN) suggested considering PHI in men with PSA 2.5–10 ng/mL, especially if previous biopsy was negative.^41^ The second, the *Four Kallikrein Assay* (4Kscore) incorporates total PSA, free PSA, intact PSA, and human kallikrein-related peptidase 2 measurements to increase detection of significant prostate cancer. These serine proteases have a differential expression in benign and cancerous prostatic tissue and can be evaluated in peripheral blood. The 4Kscore incorporates the biomarkers measured with clinical findings as well as previous biopsy. A large prospective study showed that the 4Kscore test had an AUC of 0.82 for detecting cancers with Gleason score ≥7, compared to 0.75 using total and free PSA.^42^ Eventually, NCCN recommended the 4Kscore Test for the same indications as PHI. Additional serum markers to detect aggressive prostate cancer are currently evaluated, but none has yet been incorporated in routine practice.

Additional non-serum-based markers were evaluated. The *prostate cancer antigen 3* (PCA3) *urine test* is based on the observation that mRNA of this gene was shown to be overexpressed in prostate cancer tissue almost exclusively.^43^ A PCA3 score is determined based on the ratio of PCA3 mRNA over PSA mRNA collected from a urine specimen. A published review estimated sensitivity to be 53%–84% and specificity 71%–80%. Although not perfect, PCA3 outperformed PSA and free PSA in predicting cancer detection on repeated biopsy when previous biopsy was negative.^44^ However, most papers included in the review had small sample sizes and used different criteria for biopsy referral and cut-off point to define an abnormal test. The PCA3 test may eventually lead to a reduced need for biopsy, especially if combined with modern prostate imaging, but this is yet to be proven.

The optimal time to initiate and to end screening is an additional question that must be addressed when evaluating screening programs. It is influenced by the ability to detect clinically significant disease at different ages, by life expectancy, and by national medical resources. Most current guidelines support the notion that screening should be considered for men starting at age 50 and should be offered earlier to men at high risk for prostate cancer (African Americans, men with positive family history of prostate cancer, and those who are BRCA1 or BRCA2 mutation carriers), beginning at age 40–45.^45^–^48^ These high-risk populations might benefit more from screening, although no high-level evidence is available. The 2013 American Urological Association guidelines do not advocate screening for men younger than 40, men with a life expectancy of less than 10–15 years, or men older than 70. In higher-risk men aged 40–54 and men over 70 in good health condition, screening might be beneficial and should be discussed individually. The frequency of PSA testing also remains undetermined, although baseline PSA level could guide testing intervals.

In the last few years many biomarkers have been assessed for better prediction of screening outcomes and potential clinical utility. The most commonly reported are PCA3, TMPSS2, MMP-9, ANXA3, GSTP1, BRCA1, BRCA2, mismatch repair genes, HOXB13, and HPC. Although currently standard practice does not include genetic testing for prostate cancer screening, reports of increased prostate cancer risk in men with mutations in BRCA1 and BRCA2 have been published.^49^–^54^ Increased risk has also been noted in the mismatch repair (MMR) gene mutation carriers.^55^ Clinical genetic testing is available, but its use in screening remains to be defined.

Recently BRCA1 and BRCA2 mutations have gained increased interest as several studies indicate the risk may be greater among men with the BRCA2 founder mutation than among those with one of the BRCA1 mutations.^49^ Data suggest poor survival rates in BRCA2 mutation carriers relative to BRCA1 mutation carriers.^52^ Even if not fully investigated, this should clearly reflect on individualized screening for this population.

In real life, some patients should be considered for genetic susceptibility analysis to better stratify the risk to develop prostate cancer. Patients with family history of renal, bladder, colorectal, or uterine cancer should be referred for genetic analysis.^56^ The same should apply for men with a family history of prostate, breast, ovarian, or pancreatic cancer. The American Cancer Society recommends to start screening at 45 years of age for men with a first-degree relative having prostate cancer, and at age 40 years for men with two or more first-degree relatives diagnosed. Other studies have demonstrated that men of African descent in the United States are more likely to develop prostate cancer. This suggests a combination of high-risk genetic and environment factors.^57^ Other medical conditions such as prostatitis,^58^ obesity, and metabolic syndrome^59^ were also shown to be associated with prostate cancer.

Recently, interest in syndromes involving DNA mismatch repair (MMR) gene mutation has increased. Increased prevalence of prostate cancer in members of families with Lynch syndrome has been reported. Given that the added risk is likely to be modest and the prevalence of prostate cancer is high, a literature review estimated a 2.13-fold (95% CI, 1.45–2.80) increased risk of prostate cancer for male carriers in clinic-based retrospective cohorts, and 2.28 (95% CI, 1.37–3.19) for all men from mutation-carrying families.^60^ Lynch syndrome male family members should also be considered for earlier and more frequent screening.^61^ The currently ongoing IMPACT trial aims to assess the use of PSA-based screening in BRCA1 and BRCA2 carrier Lynch syndrome patients (MSH1, MSH2, and MLH1). Preliminary results show that PPV of PSA in BRCA is high and screening detects significant tumors.^47^

Additionally, single nucleotide polymorphisms (SNPs) associated with prostate cancer have been evaluated, although the sensitivity is still unclear. Studies have indicated that the combination of several SNPs can increase the risk of developing prostate cancer,^62^,^63^ but difficulty arises in interpreting results when considering the impact on the individual patient. Trying to translate this to clinical practice, researchers have combined SNP testing with analysis of family history, age, and race to predict the risk of prostate cancer. The results have led to only marginal improvement in prediction.^64^

Taking all that information into account has led to an attempt to combine biomarkers and clinical findings to generate an alternative approach for prostate cancer screening. Grönberg et al. combined PSA, associated isoforms (e.g. free, intact, and human kallikrein 2), two plasma biomarkers (MSMB and MIC1), 232 germline SNPs, clinical variables (age, family history, and previous biopsy), and clinical findings (e.g. digital rectal examination and prostate volume) in a screening program in Sweden.^65^ Compared to using a 3 ng/mL PSA threshold for recommending a biopsy, the reported strategy effectively diagnosed all men with Gleason ≥7 while decreasing the biopsy rate by 32% (improved area under the curve from 0.56 to 0.74). A total of 104 men with Gleason 6 prostate cancer were undiagnosed by this trial, all with <10 mm of cancer. This report probably represents the future of screening, with improved performance compared to the traditional approach.

Recent advances in medical imaging have also drawn growing interest in the field of screening. Over the last several years, magnetic resonance imaging (MRI) of the prostate has been increasingly incorporated in prostate cancer active surveillance and treatment assessment. Naturally, its use for screening has also been assessed. The PROMIS group recently reported a confirmatory study testing diagnostic accuracy of multiparametric MRI (MP-MRI) and transrectal ultrasound (TRUS)-guided biopsy results versus template prostate mapping biopsy as a reference test for men with PSA up to 15 ng/mL.^66^ On template biopsy, 71% of men were diagnosed with prostate cancer and 40% had clinically significant cancer. For clinically significant cancer, MP-MRI was more sensitive (93%; 95% CI, 88%–96%) than TRUS-guided biopsy (48%; 95% CI, 42%–55%; *P*<0.01) but had reduced specificity. Based on the results, an additional 18% of cases of clinically significant cancer might be detected if biopsies were directed by MP-MRI findings. If MP-MRI is performed prior to the first prostate biopsy, it could reduce the number of biopsies by up to one-quarter, leading to decreased diagnosis of clinically insignificant prostate cancer. This should be viewed carefully, since current MRI technology could not completely take over the role of pathological evaluation of the prostate in the screening paradigm, and naturally would result in a trade-off in reducing insignificant prostate cancer detection rates at the expense of missing some cases of significant prostate cancer. Additional trials in this field are ongoing.

In summary, current urological practice advocates screening men aged 55–70 following a personalized discussion regarding the benefits and disadvantages. Optimal frequency of PSA testing is still undetermined, probably in the range of 2–4 years. Use of age- and risk factor-adjusted normograms to evaluate risk of significant prostate cancer disease is recommended. Screening for men aged 40–54 and men older than 70 years should be considered for high risk populations and for men with more than 10–15 years’ life expectancy, respectively.

## SCREENING FOR UROTHELIAL CARCINOMA

Sporadic urothelial carcinoma has been known to be associated with smoking, as well as aryl amines and other chemical carcinogen exposures. In addition, several genetic syndromes are known to be associated and to predispose to urothelial cancer. Increased risk of upper-tract urothelial cancer has been reported in *Lynch syndrome* (LS), as well as increased risk of bladder cancer.^67^,^68^ The estimated risk of urothelial cancer in LS is 5%–20%, with risk increasing in males and MSH2 mutation carriers. Screening for urothelial cancer was suggested to include urine analysis (to detect microscopic hematuria), abdominal ultrasound, urine cytological analysis, and novel urine molecular markers. Screening for urothelial cancer in LS patients has been endorsed by the Mallorca group as well as by the American Genetic Association; however, data regarding its effectiveness in reducing morbidity and mortality are lacking. A study by Myrhoj et al. attempted to assess surveillance strategy to detect urothelial cancer in Lynch syndrome families.^69^ Researchers reviewed 3,411 medical records of relatives of LS families, families that met the Amsterdam criteria I or II, or that had been suspected of LS (not fully fulfilling the criteria but suggestive of possible familial LS). In two patients (0.1%), the screening, involving testing urine cytology every second year starting at the age of 25, identified asymptomatic non-invasive low-grade bladder tumors. Subsequently, 14 out of 997 patients were diagnosed with urinary cancer over a period of 14 years. The overall sensitivity of urine cytology was calculated to be only 29%, and specificity was 96%. Eleven tumors were diagnosed in MSH2 *fa*milies. Given this, urine cytology was not recommended as a screening tool in LS individuals. Due to small numbers, no conclusions could be made regarding the MSH2 mutation population. Abdominal ultrasound has also been suggested as a screening tool in LS, but no high-level evidence is currently available. A recent review published in 2013 concluded that the risk of any urinary tract cancer in LS is low.^70^ Urinary tract tumor incidence was suggested to be higher in male MSH2-mutation carriers at age 50–70 years. Since no screening program has been properly evaluated, no data are currently available regarding the ability of any program to reduce morbidity or mortality. Given the known low sensitivity of urine cytology, it is not recommended as a solitary screening test for urothelial cancer. Computerized tomography (CT) scan and cystoscopy as screening tools in LS populations have not been evaluated. Potential morbidity resulting from radiation exposure, infections, as well as the cost, preclude them from current screening programs. In our opinion, they should be studied in selected cohorts of high-risk Lynch syndrome patients like those that carry MSH2 mutations or with a family history of urinary tract cancers.

During the early 1990s, reports have documented an association of endemic nephropathy as well as upper-tract transitional cell carcinoma (TCC) with aristolochic acid exposure.^71^ Additional reports have suggested that aristolochic acid may contaminate herbal medicine extracts, especially ones used as slimming pills, causing increased risk of upper-tract TCC.^72^ A recent summary of this topic suggested that aristolochic acid effects might last for years after the exposure, and suggested a potential benefit of upper-tract screening in people who commonly use herbal medicine.^73^ Most herbal medicine treatments available are not properly regulated to confirm the absence of aristolochic acid, and no data are available regarding the exposure needed to increase risk for upper-tract TCC. However, patients reporting significant herbal medicine use should be considered for screening.

## SCREENING IN BLADDER CANCER

In 2010, a data review addressing questions regarding bladder cancer screening was performed by the Preventive Services Task Force.^74^ In summary, the authors concluded that there was lack of high-level data regarding screening methods performance, as well as reports regarding potential harm, leading to lack of consensus regarding proper screening method. Given a relatively well-known natural history of bladder TCC, the goal of screening should mainly be to detect lesions prone to progress to muscle-invasive disease. The use of routine screening for hematuria in the general population has not been shown to alter outcomes of aggressive forms of bladder cancer, mainly due to low positive predictive value, leading to a vague recommendation regarding its use.^75^ The use of voided urine cytology was also considered. False-positive rates are relatively low, but the low sensitivity even when used for following up in bladder cancer patients makes it inappropriate for screening the general population. No studies have looked at outcome of cytologic screening on bladder TCC mortality other than in the context of occupational exposure. This high-risk population with occupational exposure is usually screened for bladder cancer using urine testing for hematuria and cytology. Among them, smokers are at an even higher risk. However, several studies failed to show that screening alters the outcome of bladder cancer even in this sub-group of patients.^76^ Obviously, carefully planned screening research in these workers is also warranted. Smoking significantly increases bladder cancer risk and might be associated with worse outcomes of treatment.^77^ Since risk of bladder cancer increases with duration and intensity of smoking,^78^ patients with smoking-related medical issues suggestive of significant exposure, such as chronic obstructive pulmonary disease (COPD), should also be considered for screening.

## SCREENING FOR KIDNEY CANCER

A screening approach was also considered for kidney cancer, given the fact that its natural history is nowadays better understood. Nephron-sparing surgery or tumor ablation are established methods of treatment, and localized disease can be cured. Ultrasound-based screening was suggested to have a major impact on renal cancer mortality^79^ and treatment cost-benefit ratio. Therefore, screening programs directed to detect kidney cancer are potentially beneficial. A meta-analysis evaluating the natural growth rate of renal masses which were not resected initially revealed mean tumor size at diagnosis of 2.6 cm and a mean growth rate of 0.28 cm in diameter per year.^80^ Another study reported that one-third of masses tend not to grow significantly over 2 years following diagnosis.^81^ In a prospective study reporting active surveillance for small renal masses for a median time of 3 years, 15% of masses were stable in size, suggesting a benign histology.^82^ In general, larger kidney masses are less prone to be benign and tend to grow faster. Also, larger malignant kidney masses are associated with shorter survival.^83^ Given the increasing practice of performing a kidney biopsy for small renal masses prior to surgical decision, an incidental finding of a renal mass might not warrant surgical excision in biopsy-proven benign lesions. However, one report suggests that most incidental asymptomatic malignant kidney cancers will progress to clinical disease within a few years.^84^ Kidney cancer incidence is influenced by sex, age, smoking history, obesity, hypertension, end-stage kidney disease, exposure to carcinogens, and family history.^85^ The potential harms related to kidney cancer screening include cost, excessive biopsies, and surgical morbidity. Shea calculated that in a high-risk population of obese male smokers, screening using CT would result in identifying one renal tumor in every 278 people screened. If estimated under- and overdiagnosis rates of about 25% are added, the number-needed-to-screen increases to 371.^85^ These numbers are expected to grow if screening is applied to the general population. Therefore, stratifying the population based on age, BMI, hypertension, and family history of known predisposing genetic abnormalities, as well as dialysis and environmental exposure (carcinogens, smoking, physical activity), was proposed for ultrasound-based screening.^85^ Optimal time to start screening was not evaluated and current outcomes are not available. This represents a shift toward acceptance of the paradigm of screening for kidney cancer in high-risk populations. However, no specific recommendations have been provided yet.

## CONCLUSION

In conclusion, screening unveils the opportunity to detect urological malignancies at an early stage with reduced morbidity and mortality. Concerns regarding potential harm still exist, given some design limitations in the small number of available randomized trials. In addition, the impact of screening on quality of life needs to be better understood before conclusions can be made. In prostate cancer, most recommendations adhere to the age range of 50–55 to consider starting PSA screening if life expectancy exceeds 10 years, and 40–45 for men with increased familial and genetic predisposition, although high-quality evidence for the benefit outweighing the possible risks, even in the latter subgroup, is lacking. Screening should be stopped at the age of 70 since no additional benefit was noted, but should be tailored to general health and life expectancy. Screening intervals of 2–4 years are usually recommended. Better risk stratification together with incorporating genetic and epigenetic testing and state-of-the-art imaging will probably take a central role in upcoming years, and produce a personalized protocol of screening for prostate cancer. Since the benefit of screening is apparent only many years after initiating a well-planned screening program, it will take years to reach conclusions. This may cause confusion, as new detection methods emerge before the older ones are fully evaluated. In view of the recent evidence, the United States Preventive Service Task Force issued a recent draft, prior to a recommendation update, suggesting that the decision whether to offer screening for prostate cancer should be individualized. They suggest a small potential benefit of reducing the chance of dying of prostate cancer and recommend individualized decision-making about screening for prostate cancer after discussion with the patients. This is expected to increase screening prevalence, presumably with the incorporation of new biomarkers and imaging to improve the detection rate of significant prostate cancer and to spare unwarranted complications. In other urological malignancies, high-quality screening is less frequently reported. Nevertheless, recent advancements in understanding the natural history of disease, and in blood and urine biomarkers, might warrant additional investigation of screening strategies for bladder and kidney cancers in high-risk populations.

## Abbreviations

BRCA gene: breast cancer gene
LS: Lynch syndrome
MMR: mismatch repair
MP-MRI: multi-parametric magnetic resonance imaging
PCA3: prostate cancer antigen 3
PHI: prostate health index
PPV: positive predictive value
PSA: prostate-specific antigen
SNP: single nucleotide polymorphism
TCC: transitional cell carcinoma.
